# Treatment of fossa navicularis strictures

**DOI:** 10.1590/S1677-5538.IBJU.2022.0067.1

**Published:** 2022-08-03

**Authors:** Aderivaldo Cabral Dias, Paulo Roberto Faria Ribeiro

**Affiliations:** 1 Hospital de Base do Distrito Federal Divisão de Urologia Reconstrutiva Unidade Urológica Brasília Brasil Unidade Urológica, Divisão de Urologia Reconstrutiva, Hospital de Base do Distrito Federal, Brasília, Brasil

## COMMENT

The article describes the authors’ experience with their technique to treat fossa navicularis strictures with ventral buccal mucosa grafts (1). They analyzed complications and outcomes in a retrospective case series of ten patients, selected from their institution's 151-patient strong database. The series included those with synchronous proximal strictures discontinuous with the distal one, i.e. skip strictures; and excluded those with Lichen Sclerosus et Atrophicus (LSA). In all but one patient the stricture resulted from either transurethral surgery or urinary catheterization. The authors achieved a high success rate (by the customary criterion of no further urethral instrumentation): Nine out of ten patients had a favorable functional and aesthetic outcome, and no complications such as urinary fistulae were reported.

Their technique is rather straightforward, with few modifications from the original description by Chowdhury at al (2). After cystoscopically ensuring that the distal stricture was limited to the fossa navicularis, a buccal mucosal graft was sutured (with 5-0 polydioxanone) to the mucosal edges of the ventrally incised urethra, with vascular support being provided by both a dartos flap and a glans spongiosum 2-layered closure. In narrower strictures, a dorsal Asopa-like (3) graft was used to augment the urethral lumen.

This paper timely addresses one of our most vexing complications: A fossa navicularis stricture after transurethral surgery or urethral instrumentation, which the practicing urologist will inevitably encounter many times during his or her career. Thus, knowledge of treatment options other than dilatation or simple ventral meatotomy/distal urethrotomy looks like sound individual policy. The latter procedures, however simple, effective and widely taught as they are, often lead to aesthetically unfavorable outcomes, which may compromise the patient-physician relationship.

Yet, the question whether the procedure should be performed by any urologist or by a specialist, that is, an experienced reconstructive urologist, has a less clear-cut answer. Although the authors claim that the operation has a short learning curve, it does require buccal mucosal harvesting and exquisite technique to handle both graft and graft bed, which may fall outside the scope of many urologists trained during the bygone “direct visual urethrotomy era”. Perhaps the new generation of urologists, trained in the ongoing “reconstructive era”, may judge themselves more apt to adopt this technique (or any of its variations that will surely follow). For those lacking proper reconstructive urology training, either during their residency or by going through a reconstructive urology fellowship, it may be wise to address the knowledge gap by referencing these patients to more experienced colleagues, and to involve oneself in these operations until acquiring familiarity with the procedure and with its postoperative care.

A subtler point of this procedure is that it may act to preserve some of the hydrodynamic features of the fossa navicularis, lost in simple ventral meatotomy/distal urethrotomy and ventrally placed penile skin flap (that are not an option in LSA patients) procedures. The fossa owes its transversal elliptical shape from the combination of the higher tissue compliance of the glans spongiosum and Henle's septum glandis (4,5), which tethers the urethra at its 6 and 12 o'clock positions, and seems to function as a urinary “flow control” chamber (6). Indeed, one may conjecture that with time the graft should accommodate to its relatively capacious glanular bed, and acquire a geometric configuration capable of producing a coherent, wave-like, normal voiding stream.
